# Development of fully automated and ultrasensitive assays for urinary adiponectin and their application as novel biomarkers for diabetic kidney disease

**DOI:** 10.1038/s41598-020-72494-6

**Published:** 2020-09-28

**Authors:** Toshihiro Watanabe, Yuki Fujimoto, Aya Morimoto, Mai Nishiyama, Akinori Kawai, Seiki Okada, Motohiro Aiba, Tomoharu Kawano, Mina Kawahigashi, Masashi Ishizu, Hiroyasu Mori, Munehide Matsuhisa, Akiko Hata, Makoto Funaki, Seiichi Hashida

**Affiliations:** 1 R&D Division, Sysmex R&D Center Americas, Inc., Mundelein, IL USA; 2 Life Style Diseases, Institute for Health Sciences, Tokushima Bunri University, Tokushima, Japan; 3 Clinical Innovation, Sysmex Corporation, Kobe, Hyogo Japan; 4 Human Life Science, Tokushima Bunri University, Tokushima, Japan; 5 Diabetes Therapeutics and Research Center, Institute of Advanced Medical Sciences, Tokushima University, Tokushima, Japan; 6 Clinical Research Center for Diabetes, Tokushima University Hospital, Tokushima, Japan; 7 Department of Diabetes and Molecular Genetics, Ehime University Graduate School of Medicine, Ehime, Japan

**Keywords:** Biomarkers, Diabetes, Kidney diseases, ELISA

## Abstract

Glomerular filtration rate (GFR) and urinary albumin excretion rate (UAER) are used to diagnose and classify the severity of chronic kidney disease. Total adiponectin (T-AN) and high molecular weight adiponectin (H-AN) assays were developed using the fully automated immunoassay system, HI-1000 and their significance over conventional biomarkers were investigated. The T-AN and H-AN assays had high reproducibility, good linearity, and sufficient sensitivity to detect trace amounts of adiponectin in the urine. Urine samples after gel filtration were analyzed for the presence of different molecular isoforms. Low molecular weight (LMW) forms and monomers were the major components (93%) of adiponectin in the urine from a diabetic patient with normoalbuminuria. Urine from a microalbuminuria patient contained both high molecular weight (HMW) (11%) and middle molecular weight (MMW) (28%) adiponectin, although the LMW level was still high (52%). The amount of HMW (32%) and MMW (42%) were more abundant than that of LMW (24%) in a diabetic patient with macroalbuminuria. T-AN (r = − 0.43) and H-AN (r = − 0.38) levels showed higher correlation with estimated GFR (eGFR) than UAER (r = − 0.23). Urinary levels of both T-AN and H-AN negatively correlated with renal function in diabetic patients and they may serve as new biomarkers for diabetic kidney disease.

## Introduction

Chronic kidney disease (CKD) is a disease, in which chronic renal impairment or decline in renal function is followed by an end-stage renal disease that requires dialysis or a kidney transplant^1^. In addition, CKD has been shown to increase the risk of cardiovascular disease (CVD) such as myocardial infarction, stroke, and heart failure, as well as death^2^. Therefore, early diagnosis and appropriate treatment of CKD is critical to prevent the onset/progression of CKD and the development of CVD. Glomerular filtration rate (GFR) and urinary albumin excretion rate (UAER) have been used to diagnose and classify the severity of CKD^3^, and previous studies have shown that urinary adiponectin may be useful as a biomarker of CKD, as well.

Several studies have confirmed that urinary adiponectin increases with the progression of renal impairment in patients with diabetes^4–6^. It has also been reported that predicting the onset/progression of such impairments by either GFR or UAER is difficult, but urinary adiponectin levels may enable this prediction^7,8^. Furthermore, increased urinary adiponectin has been reported in IgA nephropathy^9^ and in nephropathy caused by systemic lupus erythematosus^10,11^. Urinary adiponectin has been thought to be excreted due to either disruption of the glomerular barrier^8–10^, tubular injuries^8,12^ or vascular damages^13^, and it is of interest to investigate if urinary adiponectin would have significance as a biomarker over other conventional biomarkers in diabetic kidney disease (DKD).

Immunoassay technology is widely used for detection of protein biomarkers and novel technologies are continuing to be developed^14–21^. Adiponectin is present in urine at very low concentrations (pg/mL levels)^4–13^ and a commercial immunoassay kit do not have sufficient analytical sensitivity to detect these trace amounts^10^. In recent years, an ultrasensitive immunoassay system capable of measuring down to a concentration of pg/mL or less has been put into practical use^22,23^. Immune complex transfer enzyme immunoassay (ICT-EIA) ^24^, which is one of the methods to realize ultrasensitive immunoassay detection, can be adapted to high-throughput immunoassay systems used in clinical settings^25–28^. Using antibody-coated magnetic particles to increase capture efficiency, immune complex transfer enables interfering background signal to be eliminated, thereby enhancing analytical sensitivity.

Previously, we developed a manual ICT-EIA to detect trace amounts of total adiponectin (T-AN) in urine^29^.We have also reported that patients with diabetes show increased T-AN levels in their urine, in spite of a decrease in their blood, when compared with healthy and obese subjects. Analysis of urine by gel filtration revealed that urine T-AN consists of four isoforms, which are high molecular weight (HMW), medium molecular weight (MMW), low molecular weight (LMW), and monomers. HMW adiponectin (H-AN) is increased in the urine of a patient with microalbuminuria compared with that in the urine of a patient with normoalbuminuria. Furthermore, T-AN concentrations, but not UAER, shows a significant correlation with eGFR. As a result, urinary adiponectin may be a useful surrogate marker for the decline of eGFR.

As described above, the molecular morphology of urinary adiponectin may be altered in response to the progression of DKD. Therefore, we developed a method, which is capable of differentially quantifying all adiponectin isoforms, which is sensitive enough to detect trace amounts of them found in the urine. T-AN assays, which we reported previously^29^, was performed manually with multiple steps. Thus, it is complicated and time consuming, and it may be challenging to conduct in clinical situations. In this paper, we demonstrate our fully automated ultrasensitive T-AN assay and a new H-AN assay, which is also ultrasensitive and fully automated. Furthermore, we show the significance of these two assays as new biomarkers for DKD.

## Results

### Reactivities of T-AN and H-AN assays to adiponectin multimers and monomer fractions

**Figure 1:**
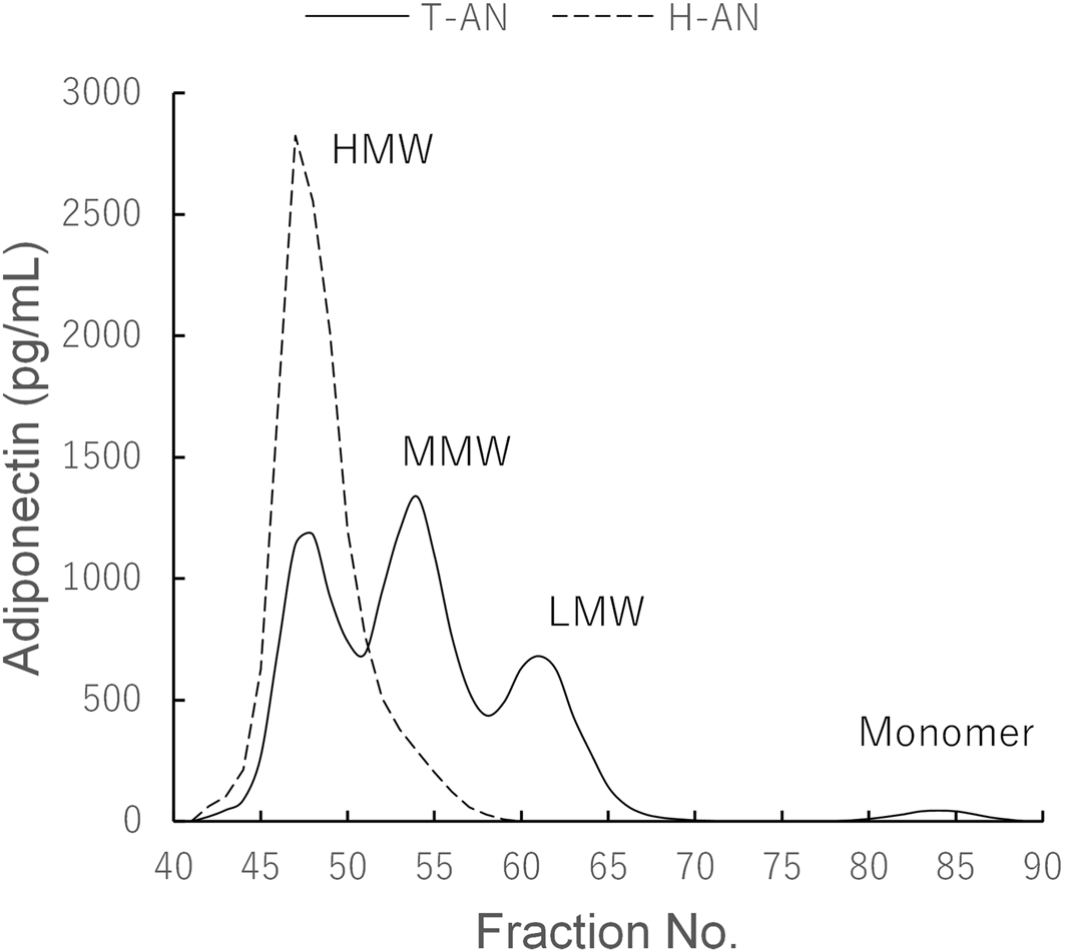
Reactivities of T-AN and H-AN assays to multimers and monomer fractions of urinary adiponectin. SEC fractions of a urine sample from a patient with macroalbuminuria were analyzed with T-AN and H-AN assays.

A urine sample obtained from a patient with macroalbuminuria was fractionated using size exclusion chromatography (SEC) and fractions were analyzed using both T-AN and H-AN assays (Fig. 1). Similar to our previous study, in which T-AN assays were conducted manually^29^, 4 peaks, which correspond to HMW, MMW, LMW and monomeric adiponectin, respectively, were observed by the T-AN assay. In contrast, the H-AN assay generated a single peak that corresponded to HMW adiponectin, although a weak signal without a peak was also detected in fractions corresponding to MMW adiponectin. However, the MMW signal is remarkably low compared to the signal corresponding to HMW, demonstrating the specificity of our H-AN assay for reporting the amount of HMW in the urine.

### Analytical performance of T-AN and H-AN assays

**Figure 2:**
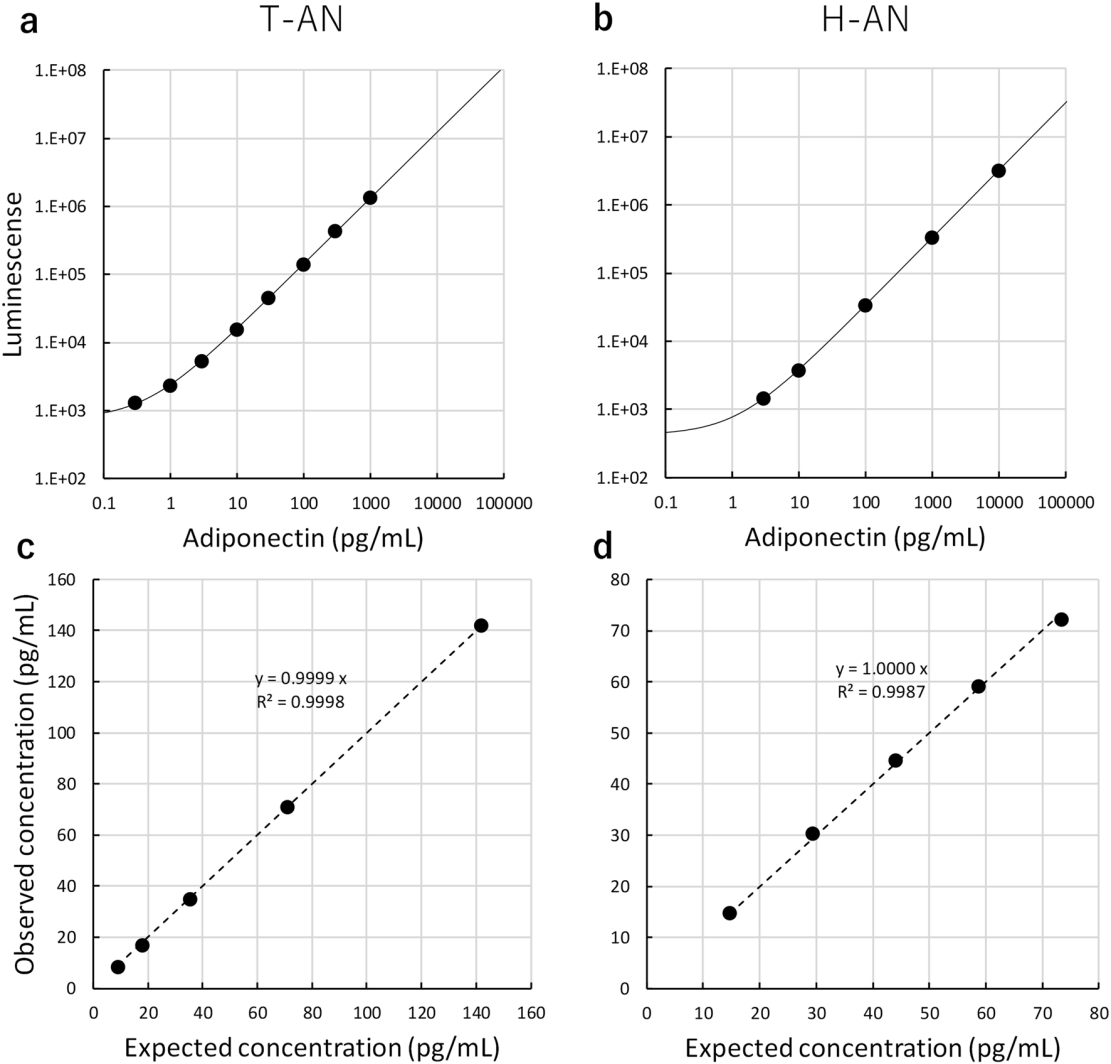
Analytical performance of T-AN and H-AN assays. Dose and response curves for T-AN (**a**) and H-AN (**b**) assays. Dilution linearity of T-AN (**c**) and H-AN (**d**) assays.

Analytical performance of T-AN and H-AN assays are shown in Fig. 2. Dose and response curves were obtained for these two assays (Fig. 2a,b) and LoDs (Limits of Detection) were calculated as 0.05 and 0.11 pg/mL for T-AN and H-AN assays, respectively, using the mean plus 3SD of low concentration samples. Reproducibility was good with CVs of 1.8–2.6% and 2.1–5.1% (n = 10) for T-AN and H-AN assays, respectively. In testing of serially diluted samples (Fig. 2c,d), the ratios of the observed concentrations to the expected concentrations based on the dilution factor in the T-AN and H-AN assays were 93–100% and 98–103%, respectively.

**Figure 3:**
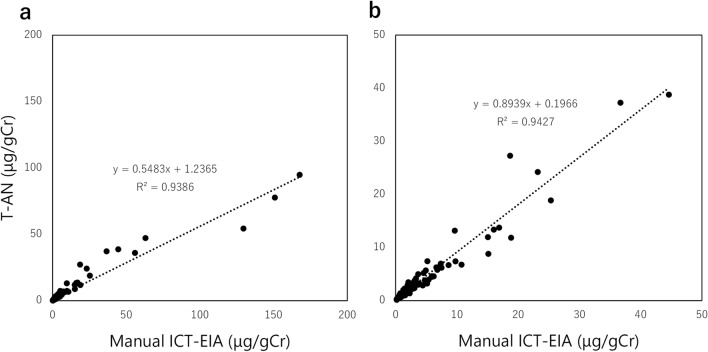
Correlation between two assays for T-AN levels in the urine; a manual assay (Manual ICT-EIA) and a fully automated T-AN assay on HI-1000 (T-AN.) (**a**) All samples, (**b**)samples with a measurement of T-AN levels by the manual ICT-EIA < 50 μg/gCr.

A manual ICT-EIA for T-AN was previously developed and a feasibility study of urinary T-AN levels as a CKD biomarker was reported^29^. The newly developed T-AN assay uses the same antibody pair, and has a comparable sensitivity with the manual ICT-EIA, while the assay time was shortened from 3 days to 1 h because of the fully automated process on HI-1000. A measurement by the manual ICT-EIA and a measurement by the T-AN assay on HI-1000 showed a strong correlation (Fig. 3). However, for samples containing high levels of T-AN (above 50 μg/gCr), the measurement on HI-1000 tended to be lower than that by the manual ICT-EIA (Fig. 3a). When those samples were excluded, the slope was close to 1 (Fig. 3b).

### Composition of adiponectin multimers in the urine of DKD patients

**Figure 4:**
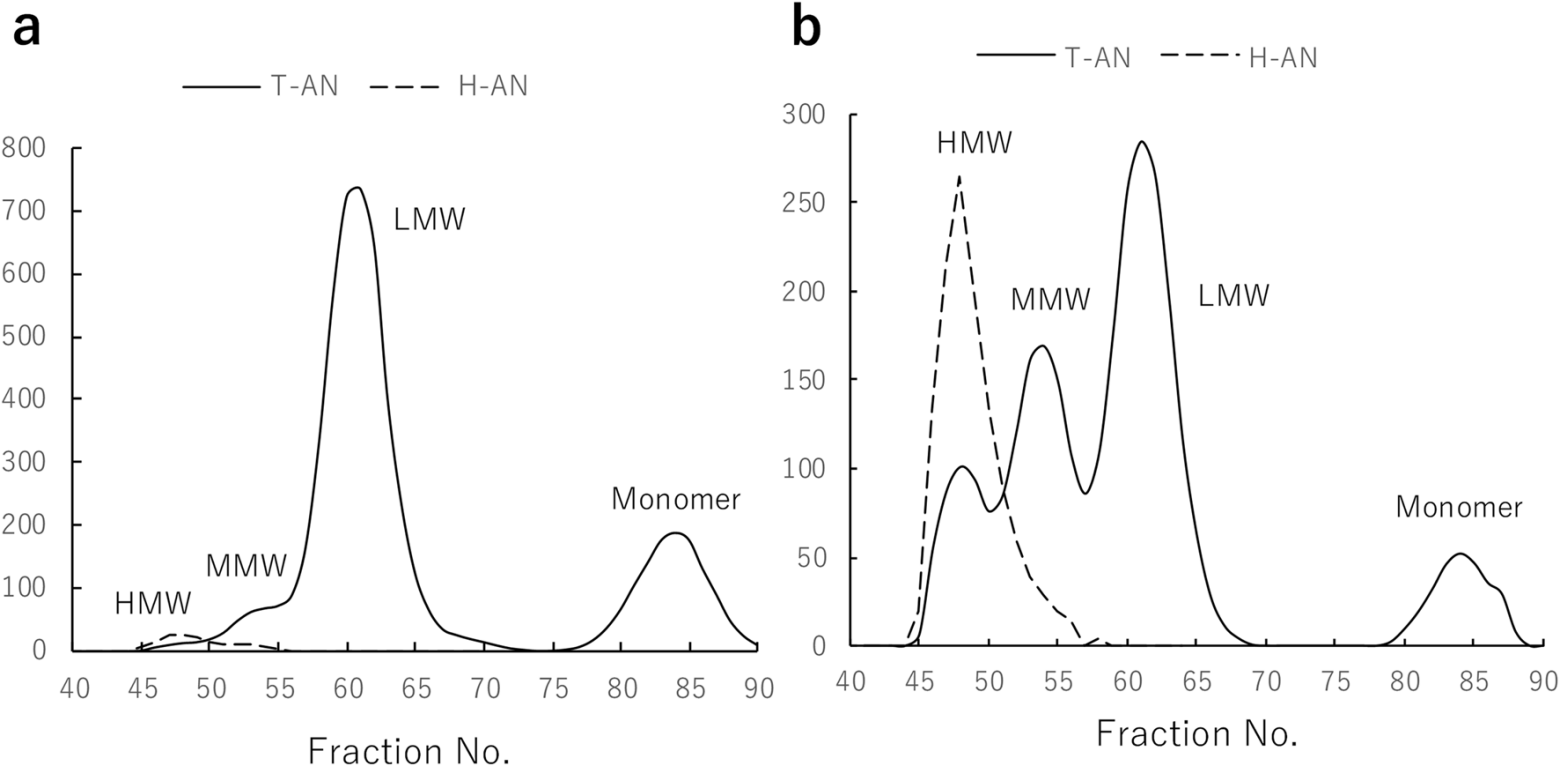
Composition of adiponectin in urine from patients with normoalbuminuria (**a**) and microalbuminuria (**b**). Urine samples from a patient with normoalbuminuria and a patient with microalbuminuria were separated by SEC and fractions were measured with T-AN and H-AN assays.

In addition to the data of a patient with macroalbuminuria shown in Fig. 1, urine samples from a patient with normoalbuminuria and a patient with microalbuminuria were also fractionated by SEC and assayed for T-AN, H-AN, albumin and creatinine (Fig. 4) and the background data for these 3 cases are shown in Table 1. Both T-AN and H-AN levels were higher in the urine of patients with either microalbuminuria or macroalbuminuria, which could be mainly attributed to the appearance of HMW and MMW in the urine of those patients.

**Table 1:** Urinary biomarker concentrations in diabetic patients with normoalbuminuria, microalbuminuria, and macroalbuminuria shown in Figs. 1 and 4.

	Albuminuria		
	Normo (Fig. 4a)	Micro (Fig. 4b)	Macro (Fig. 1)
Albumin (mg/gCr)	7.5	83	2926
T-AN (μg/gCr)	5.7	19	78
H-AN (μg/gCr)	0.14	8.6	78
eGFR (mL/min/1.73m^2^)	100	34	43

### Relationship of T-AN and H-AN levels with DKD severity

Urine samples from 77 patients with either type 1 or type 2 diabetes and 97 non-diabetic controls were tested for T-AN, H-AN, albumin and creatinine. Characteristics of these two groups are summarized in Table 2. In this study, the non-diabetic subject group was a younger population (42, 34–49; Median, 25th-75th percentiles) than the diabetic patient group (62, 52–69), which might affect the levels of urinary biomarkers. Thus, a subgroup of 42–64 years old only was also investigated. The age of inclusion criteria in the analysis of 42–64 years was determined by the maximum age of non-diabetic subjects and the minimum age at which no statistical difference of age between the groups was observed.

**Table 2:** Characteristics of non-diabetic and diabetic groups.

All subjects	Non-diabetic subjects (n = 97)	Patients with diabetes (n = 77)	*p*
Gender (Male/female)	64/33	38/39	
Age (years)	42 (34, 49)	62 (52, 69)	< 0.001
BMI (kg/m^2^)	24 (21, 26)	24 (22, 28)	0.098
Type of diabetes (1/2)	N/A	31/46	
Duration of diabetes (years)	N/A	12 (7.5, 18)	
HbA1c (%)	5.4 (5.2, 5.6)	7.1 (6.7, 7.7)	< 0.001
eGFR (mL/min/1.73 m^2^)	88 (76, 96)	68 (55, 83)	< 0.001
Urinary albumin (mg/gCr)	2.3 (1.5, 3.5)	4.2 (2.1, 35)	< 0.001
T-AN (μg/gCr)	0.89 (0.63, 1.2)	3.8 (1.4, 9.6)	< 0.001
H-AN (μg/gCr)	0.040 (0.020, 0.063)	0.21 (0.060, 1.5)	< 0.001

42–64 years old only	Non-diabetic subjects (n = 49)	Patients with diabetes (n = 41)	*p*
Gender (Male/Female)	32/17	20/21	
Age (years)	49 (44, 56)	54 (46, 59)	0.083
BMI (kg/m^2^)	24 (21, 27)	25 (22, 29)	0.38
Type of diabetes (1/2)	N/A	20/21	
Duration of diabetes (years)	N/A	9 (5.0, 16)	
HbA1c (%)	5.5 (5.2, 5.8)	7.2 (6.8, 8.1)	< 0.001
eGFR (mL/min/1.73 m^2^)	79 (69, 90)	77 (60, 88)	0.11
Urinary albumin (mg/gCr)	2.4 (1.7, 3.9)	3.3 (1.9, 36)	0.011
T-AN (μg/gCr)	1.1 (0.66, 1.8)	2.5 (1.2, 6.5)	< 0.001
H-AN (μg/gCr)	0.043 (0.020, 0.074)	0.11 (0.051, 0.57)	< 0.001
	Median (25%, 75%)		

For comparison of differences between two groups, the Mann–Whitney's U test was used.

Spearman's rank correlation coefficients between conventional biomarkers and T-AN or H-AN, all of which were normalized by creatinine, using all subjects’ data are summarized in Table 3. T-AN and H-AN levels were strongly correlated with the albumin level in subjects with/without diabetes. In addition, the correlation between the T-AN level and eGFR and between the H-AN level and eGFR were stronger than that of the albumin level and eGFR especially in patients with diabetes. The same trends were observed in the age-restricted subgroup of 42–64 years old only (Table S2).

**Table 3:** Correlation between conventional biomarkers and T-AN or H-AN in the urine.

	ND	DM	ND + DM
**Albumin**			
T-AN			
r	0.25	0.70	0.54
p	0.014	< 0.001	< 0.001
H-AN			
r	0.47	0.75	0.66
p	< 0.001	< 0.001	< 0.001
**eGFR**			
Albumin			
r	0.013	− 0.32	− 0.23
p	0.90	0.0048	0.0025
T-AN			
r	− 0.079	− 0.39	− 0.43
p	0.44	< 0.001	< 0.001
H-AN			
r	0.0017	− 0.49	− 0.38
p	0.99	< 0.001	< 0.001

ND, Non-diabetic subjects; DM, Type 1 and type 2 diabetes patients. Spearman's correlation coefficients were calculated.

**Figure 5:**
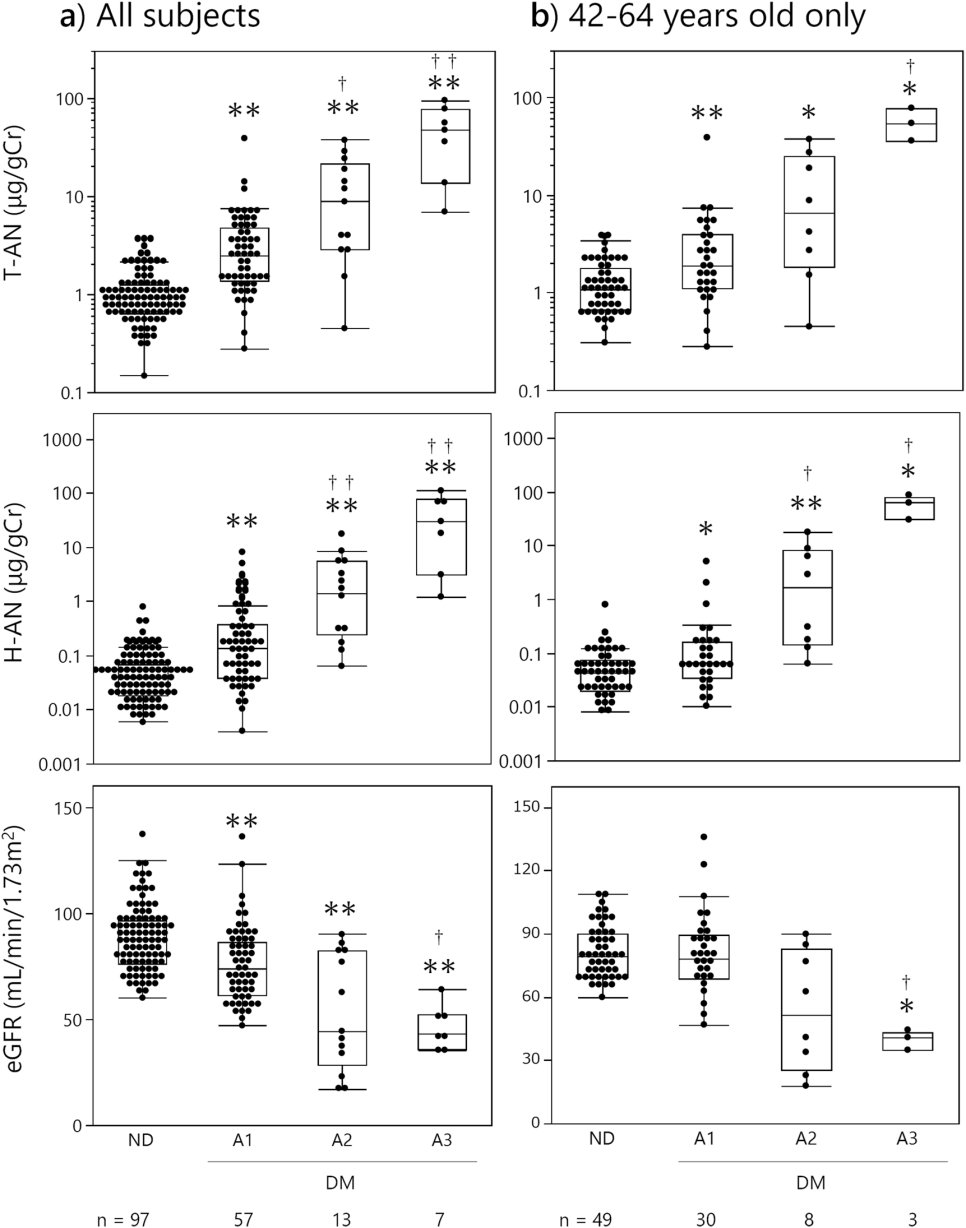
Levels of urinary biomarkers and eGFR at each albumin stage for all subjects (**a**) and subjects aged 42–64 years old only (**b**). Albumin stages were classified according to the following definition. A1: < 30, A2: 30–299, A3: ≧ 300 mg/gCr. Kruskal–Wallis test and Steel–Dwass test were used for comparisons. ND: Non-diabetic subjects, DM: patients with Type 1 or type 2 diabetes, *P < 0.05 and **P < 0.01 compared with ND, ^†^*P* < 0.05 and ^††^*P* < 0.01 compared with A1.

Levels of urinary biomarkers and eGFR at each albumin stage for all subjects are shown in Fig. 5a. Both T-AN and H-AN levels increased and eGFR decreased as the albumin stage progressed. Compared to non-diabetic subjects, T-AN and H-AN levels were increased significantly in diabetic patients of stage A1, A2, and A3. Similarly, T-AN and H-AN were elevated in stage A2 and A3 compared to stage A1. And further, T-AN and H-AN were elevated in A3 compared to A2. The level of eGFR was also significantly decreased in stages A1, A2, and A3 compared with non-diabetic controls and a significant decrease was confirmed at stage A3, but not A2, compared to A1.

In the analysis of the age-restricted subgroup of 42–64 years old only (Fig. 5b), similar increases in T-AN and H-AN levels were observed over non-diabetic subjects, increasing from A1 to A2 to A3 stages. Elevations were significant in all albumin stages over non-diabetic subjects. Further, H-AN levels were significantly elevated in stages A2, A3 over A1. But for T-AN, only stage A3 was significantly elevated over stage A1. For eGFR, only stage A3 showed a significant decrease from non-diabetic subjects or albumin stage A1 subjects.

**Figure 6:**
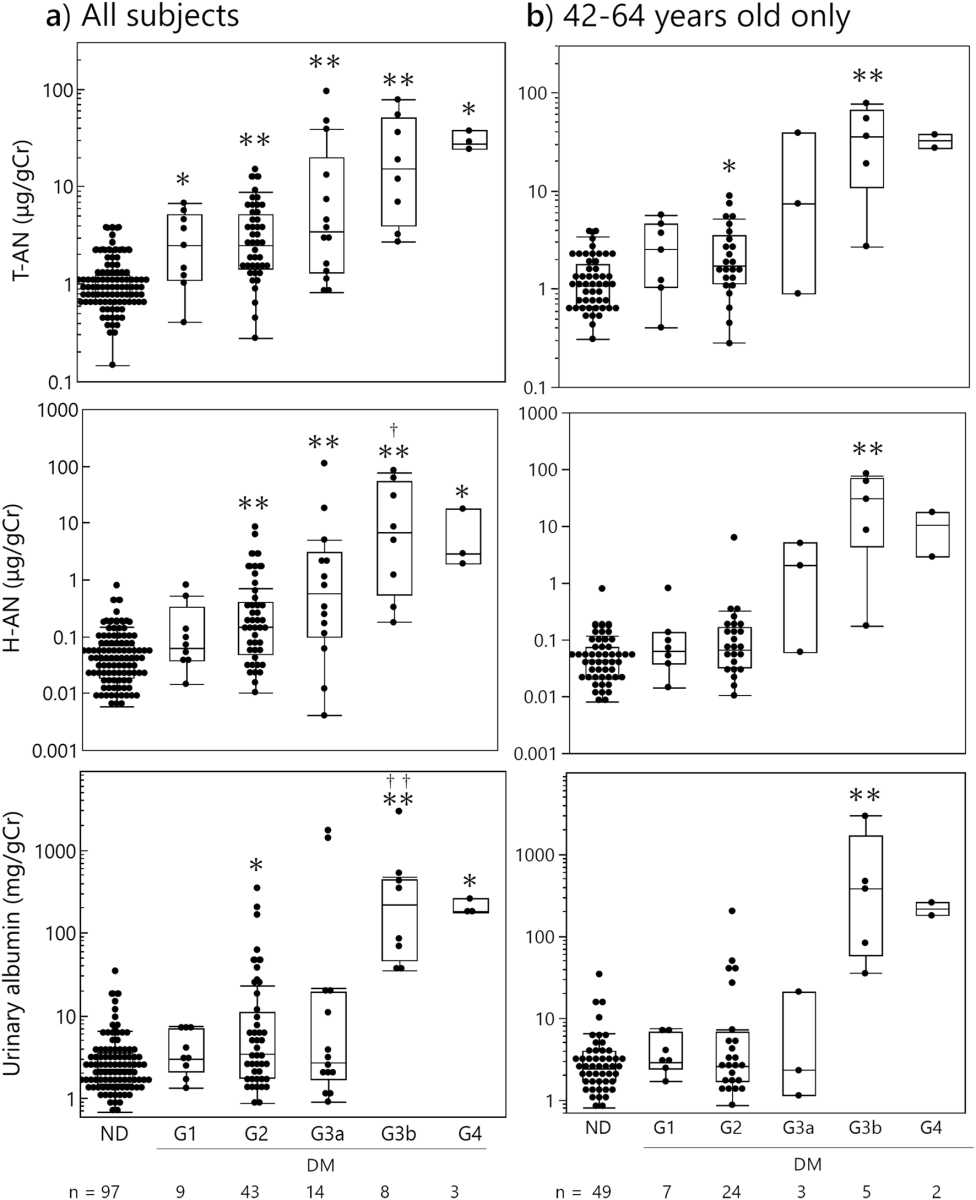
Levels of urinary biomarkers at each eGFR stage for all subjects (**a**) and subjects aged 42–64 years old only (**b**). eGFR stages were classified according to the following definition. G1: ≧ 90, G2: 60–89, G3a: 45–59, G3b: 30–44, G4: 15–29 ml/min/1.73m^2^. Kruskal–Wallis test and Steel–Dwass test were used for comparisons. ND: Non-diabetic subjects, DM: patients with Type 1 or type 2 diabetes, *P < 0.05 and **P < 0.01 compared with ND, ^†^*P* < 0.05 and ^††^*P* < 0.01 compared with G1.

Similar data analysis was performed based on eGFR stage in all subjects (Fig. 6a). Both T-AN and H-AN levels increased significantly as the eGFR stage progressed compared to non-diabetic subjects, with the exception of the H-AN levels of the G1 group which were not elevated. Albumin levels were increased significantly at eGFR stages G2, G3b, and G4 over non-diabetic subjects. Using G1 stage as baseline, no significant increase in T-AN was observed in progressive eGFR stages since the level of T-AN was already significantly high at the G1 stage. For H-AN, stage G3b levels were significantly elevated over G1 levels and this was observed also for albumin.

In the analysis of the age-restricted subgroup of 42–64 years old only (Fig. 6b), albumin and H-AN levels were statistically significantly increased, compared with non-diabetic subjects, at stage G3b whereas T-AN was statistically significantly increased in stages G2 and G3b. Similar to the results from all participants, T-AN and H-AN levels in the stage G3a and T-AN, H-AN and albumin levels in the stage G4 were high, but the small number of patients prevents statistical significance to be achieved.

Whereas T-AN and H-AN levels were strongly associated with albumin stage, higher T-AN levels were observed at earlier stages of eGFR than was albumin. These results indicate that T-AN and H-AN may be useful as early biomarkers for DKD, although the response of T-AN and H-AN levels to DKD severity is slightly different.

## Discussion

We developed assays for measuring the amount of two adiponectin isoforms; one for T-AN levels and another for H-AN levels. To measure T-AN levels, two different monoclonal antibodies, both of which recognize the globular domain of adiponectin, were used to detect all types of adiponectin. In another assay, clone 38 antibody, which detects only HMW and MMW adiponectin (Fig.S1), was used as both capture and detection to react only with HMW, and weakly with MMW adiponectin. One of the strengths of our T-AN and H-AN assays is that they are fully automated on an immunoassay system, HI-1000. As a result, the measurement time was shortened from three days for the conventional manual ICT-EIA to approximately one hour. Our fully automated T-AN and H-AN assays also show high reproducibility, good dilution linearity, and ultra-high sensitivity. In fact, the LoDs were 0.05 and 0.11 pg/mL for T-AN and H-AN assays, respectively, which would enable accurate measurement of very low levels of adiponectin in the urine of non-diabetic subjects (Fig. 5). Previous studies on urinary adiponectin used multiple commercially available Enzyme-linked immunosorbent assay kits^4,7,8,10^. These assays may differ in their reactivity to multiple isoforms of adiponectin, and reactivities of these assays to urinary adiponectin isoforms are still unknown. In this study, we developed two highly reproducible and sensitive assays that are fully automated, and have been characterized in detail with respect to the reactivity of these assays to all urinary adiponectin isoforms using SEC fractions of urine samples (Figs. 1 and 4).

Previous studies conducted by western blotting analysis have identified monomers, LMW and MMW adiponectin in urine^6,13^. Our ultrasensitive manual ICT-EIA could identify four types of adiponectin (HMW, MMW, LMW and monomer) by measurement of urine samples after SEC^29^. As described above, the fully automated and newly developed T-AN and H-AN assays are also highly sensitive enough to measure extremely low concentrations of each adiponectin isoform in urine samples after SEC (Figs. 1 and 4). Thus, it was possible to compare the level of each adiponectin isoform in urine between three patients with diabetes, who have different stages of albuminuria, by using SEC samples of urine from them. Consistent with previous reports^29^, four types of adiponectin (HMW, MMW, LMW and monomer) were detected. Moreover, the results suggested that most urinary adiponectin in patients with normoalbuminuria are monomer and LMW isoform, whereas the proportion of macromolecules, such as HMW and MMW adiponectin, are higher in patients with albuminuria (Table 1 and Fig. 4). Several mechanisms of adiponectin excretion into the urine have been proposed in the past^8–10,12,13^. Detection of more macromolecular adiponectin in patients with albuminuria supports a possibility that disruption of the glomerular molecular barrier may substantially contribute to an increased urinary adiponectin excretion in DKD.

Furthermore, we also showed that T-AN and H-AN levels were moderately to highly correlated with UAER and eGFR, which indicates that they may be used as biomarkers to tell the progression of DKD (Table 3). In fact, when T-AN and H-AN levels were measured in non-diabetic subjects and diabetic patients with various levels of DKD, T-AN level in diabetics became significantly higher than that in non-diabetic subjects from as early as the G1 stage (Fig. 6). A similar trend was observed with the age-restricted subgroup. H-AN levels showed a similar trend to albumin, with elevations observed in diabetics with > G2 stage when compared with those in non-diabetics. Thus, T-AN and H-AN levels may serve as biomarkers for DKD: usage of T-AN assay to detect a wide variety of adiponectin isoforms may enable detection of early pathological changes in the kidney. On the other hand, H-AN levels could be a biomarker of more progressed DKD.

In conclusion, we developed two ICT-EIAs for measuring T-AN and H-AN which showed high reproducibility, good dilution linearity, and ultra-high sensitivity. The conventional ICT-EIA method took 3 days to test samples, but the newly developed assays are fully automated and can finish an assay in 1 h by using a high-sensitivity immunoassay system, the HI-1000. Furthermore, using these new assays, we showed that both T-AN and H-AN levels in the urine may be increased in patients with diabetes and they may have clinical significance in tackling DKD.

The limitations of this study are limited clinical data, cross-sectional studies, age-biased controls, and lack of accurate diagnosis of diabetic renal disease, which could be made by a biopsy. In the future, the usefulness of urinary adiponectin levels as a biomarker of DKD should be tested in larger prospective studies, which is currently underway.

## Methods

### Urinary adiponectin assay

#### Buffers

10 mM sodium phosphate buffer (pH 7.0) containing 0.1 M sodium chloride (NaCl), 1.0 mM magnesium chloride (MgCl_2_), 0.1% sodium azide (NaN_3_) and 0.1% bovine serum albumin (BSA) was used for dialysis and dilution of urine samples. 0.1 M 2-(N-morpholino)ethanesulfonic acid (MES) buffer (pH 6.5) containing 0.15 M NaCl, 1.0 mM MgCl_2_, 0.1 mM zinc chloride (ZnCl_2_), 0.1% NaN_3_ and 0.1% BSA was used for dilution of detection antibody conjugates. The same composition without MgCl_2_ and ZnCl_2_ was used for dilution buffer for capture antibody conjugates. 0.1 M MES buffer (pH 6.5) containing 2.5 mM Nε-DNP-L-lysine hydrochloride (DNP), 2% casein sodium and 0.1% NaN_3_ was used for elusion buffer in ICT-EIA. HISCL washing solution (Sysmex, Hyogo, Japan) was used for washing buffer.

#### Antibodies and antigens

Different types of antibodies were employed to measure T-AN levels and H-AN levels, respectively. Monoclonal mouse anti-human Adiponectin/Acrp30 antibody (Product code: MAB10651, Clone: 166126, Antibody Registry: AB_2221612) and monoclonal mouse anti-human Adiponectin/Acrp30 antibody (Product code: MAB1065, Clone: 166128, Antibody Registry: AB_2273512) were chosen as capture and detection antibodies, respectively, for T-AN assay. Monoclonal mouse anti-human Adiponectin/Acrp30 antibody (Clone: 38, Sysmex, Hyogo, Japan) was used for both capture and detection antibodies for H-AN assay. Recombinant Human Adiponectin (Oriental yeast, Tokyo, Japan) was used as calibrators.

#### Preparation of capture and detection antibody conjugates

For T-AN assays, thiol groups were introduced into the capture antibody (Clone: 166126) using PIERCE SATA (N-succinimidyl S-acetylthioacetate) (Thermo Fisher Scientific Inc., Waltham, MA) and the antibody was then conjugated with 6-maleimidohexanoyl-DNP and 6-maleimidohexanoyl-biocytin^30,31^. Other monoclonal antibodies were digested with pepsin (Roche, Basel, Switz) to F(ab’)2, which was further reduced to obtain Fab’. Fab’ fragments were conjugated with 6-maleimidohexanoyl-DNP-biotinyl-BSA as capture antibody conjugate and with alkaline phosphatase from calf intestine (Oriental yeast, Tokyo, Japan) using N-(ε-maleimidocaproyloxy)succinimide (Dojindo, Kumamoto, Japan) as detection antibody conjugate^30,31^.

#### Preparation of protein-coated magnetic particles

Carboxyl groups on their surface of magnetic particles (JSR, Tokyo, Japan) were activated by using 1-Ethyl-3-(3-dimethylaminopropyl)carbodiimide, hydrochloride (Dojindo, Kumamoto, Japan) and N-Hydroxysuccinimide (Tokyo Chemistry Industry, Tokyo, Japan). The activated magnetic particles were mixed and coated with monoclonal mouse anti-DNP antibody (Clone: 1853, Sysmex, Hyogo, Japan) and Biotinyl-BSA, respectively.. Biotinyl-BSA-coated magnetic particles were then reacted with streptavidin (MilliporeSigma, St.Louis, MO)^30,31^.

#### Immune complex transfer enzyme immunoassay (ICT-EIA) for adiponectin

**Figure 7:**
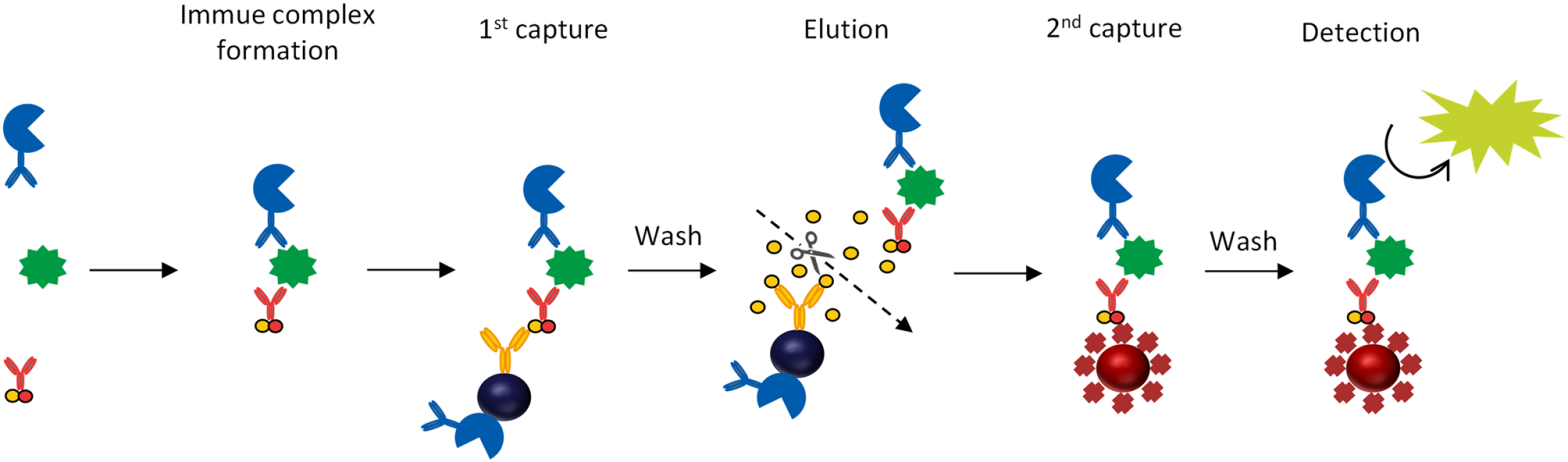
Assay scheme of ICT-EIA. All reactions were performed on HI-1000. Immune complexes formed in the liquid phase are captured by anti-DNP antibody beads. After washing, the immune complex is released into the liquid phase by the addition of large amounts of DNP-Lys and re-captured by streptavidin beads. After a second wash, the amount of chemiluminescence produced by the enzyme–substrate reaction is measured^24^.

The protocol of the ICT-EIA for T-AN was as follows (Fig. 7): 70 μL of sample was mixed with 40 μL of detection antibody conjugate and incubated for 3.4 min. 40 μL of capture antibody conjugate was added and incubated for 23.8 min. After adding 20 μL of anti-DNP antibody coated magnetic particles, immune complexes were captured onto the surface of the beads during 11.3 min of incubation. After washing, the immune complexes were released from the beads surface during 4.6 min of incubation in 110 μL of elution buffer. With magnetic particles magnetized, 80 μL of the supernatant of the reaction solution containing the immune complexes was taken and transferred to another vial. After adding 30 μL of streptavidin coated magnetic particles, immune complexes were re-captured onto the surface of the beads during 5.3 min of incubation. All reactions up to this point were performed at 37 °C. The bead was then washed, and the bound alkaline phosphatase activity was assayed by chemiluminescence with HISCL substrate reagent set (Sysmex, Hyogo, Japan) for 5.0 min at 42 °C. H-AN was measured in a similar manner, except that the reaction time with the detection antibody conjugate was 5.8 min and with the capture antibody conjugate was 9.4 min. All reactions were performed automatically using a high-sensitivity immunoassay system, the HI-1000 (Sysmex, Hyogo, Japan).

#### Assay characterization

Calibration curves were generated with 9 calibrators spanning a dynamic range of 0 to 1000 pg/mL for T-AN assay and 6 calibrators spanning a dynamic range of 0 to 10,000 pg/mL for H-AN assay. The LoD was calculated as the concentration which is equivalent to the mean plus 3 standard deviations of low concentration samples. The calibrators were measured 10 times to determine reproducibility, and coefficients of variations were calculated. In a dilution linearity evaluation, a dialyzed urine sample was diluted with dilution buffer to achieve 4 new concentrations. These 4 dilutions and the original calibrator were measured 3 times, respectively, and the difference between the concentration of the original sample and the expected concentrations calculated from the concentration of the diluted samples and their dilution factors were compared.

## Clinical study

### Subjects and samples

Nighty seven non-diabetic subjects from an occupational-based cohort study held in Tokushima prefecture and 77 outpatients with type 1 or type 2 diabetes in Tokushima University Hospital were enrolled in this study. Non-diabetic subjects were defined as satisfying the following three points: fasting blood glucose level was less than 126 mg/dl, HbA1c was less than 6.5%, and no diabetes medication was prescribed. Diabetes was defined by physicians according to the diagnostic criteria of Japan Diabetes Society^32^. Blood samples were drawn from an antecubital vein of subjects. Early morning urine samples were collected after overnight fasting. The urine samples (10 mL) were mixed with 0.1 mL of 10% BSA and NaN_3_ and dialyzed against dialysis buffer at 4 °C overnight. Dialyzed urine samples were kept frozen at -30 °C until analysis.

### Size exclusion chromatography (SEC) of urine

Three randomly chosen urine samples (1.0 mL) from patients with normoalbuminuria, microalbuminuria and macroalbuminuria, respectively, were selected from patients with diabetes and separated by SEC using an AKTA explorer 10S (GE Healthcare, Tokyo, Japan) on a column of HiLoad 16/60 Superdex 200 prep grade (1.6 × 60 cm) (GE Healthcare). An aliquot of each fraction was used for T-AN and H-AN assays.

### Measurements of biomarkers in urine

Dialyzed urine samples were analyzed for adiponectin, using T-AN and H-AN as described above. They were also analyzed for albumin, by an ICT-EIA for albumin^29^ and creatinine by a commercially available creatinine kit (Creatinine-test Wako, FUJIFILM Wako Pure Chemical Corporation, Osaka, Japan). Urinary adiponectin and albumin concentrations were normalized by the urinary creatinine (Cr) concentration. Urine samples after SEC fractionation were also tested for T-AN and H-AN.

### Statistical analysis

The Shapiro–Wilk test was used to examine whether the measurements of biomarkers were normally distributed. For biomarker correlation analysis, Spearman's correlation coefficients were calculated. For comparison of differences between two or multiple groups, the Mann–Whitney's U test or the Kruskal–Wallis test and Steel–Dwass test were used, with 5% as the criterion for significance.

### Ethical considerations

The study protocol was approved by the ethical committee of Tokushima Bunri University (No. H29-17) and by the ethical committee of Tokushima University Hospital (No. 2894–1 for diabetics and No. 3087 for non-diabetics); all diabetic participants gave written informed consent. Non-diabetic participants were participants of an occupational-based cohort study, which was also approved by the ethical committee of Tokushima University Hospital (No. 662), and gave written informed consent to this cohort study. In No. 3087, non-diabetic participants were provided an easy mode to opt-out by a hand-out and online so that participants are well-informed about this study and their opportunity to indicate refusal. All methods were performed in accordance with the relevant guidelines and regulations.

## Supplementary information

Supplementary Information.

## Data Availability

The datasets generated during and/or analyzed during the current study are available from the corresponding author on reasonable request.
