# Supplementary material for: Development of fully automated and ultrasensitive assays for urinary adiponectin and their application as novel biomarkers for diabetic kidney disease
Source: Sci Rep. 2020 Sep 28;10:15869. doi: 10.1038/s41598-020-72494-6 (PMC7522970; doi:10.1038/s41598-020-72494-6)
Supplement: Supplementary file 1 — Supplementary Information. [file 41598_2020_72494_MOESM1_ESM.docx]

**Supplementary information**

**Development of fully automated and ultrasensitive assays for urinary adiponectin and their application as novel biomarkers for diabetic kidney disease**

Toshihiro Watanabe^1*^, Yuki Fujimoto^2^, Aya Morimoto^3^, Mai Nishiyama^3^, Akinori Kawai^3^, Seiki Okada^3^, Motohiro Aiba^2^, Tomoharu Kawano^4^, Mina Kawahigashi^4^, Masashi Ishizu^5^, Hiroyasu Mori^5^ Munehide Matsuhisa^5^, Akiko Hata^6^, Makoto Funaki^6^ and Seiichi Hashida^7^

1. R&D division, Sysmex R&D Center Americas, Inc., Mundelein, IL, America, [Watanabe.Toshihiro@sysmex.co.jp](mailto:Watanabe.Toshihiro@sysmex.co.jp)
2. Life Style Diseases, Institute for Health Sciences, Tokushima Bunri University, Tokushima, Japan
3. Clinical Innovation, Sysmex Corporation, Kobe, Hyogo, Japan
4. Human Life Science, Tokushima Bunri University, Tokushima, Japan
5. Diabetes Therapeutics and Research Center, Institute of Advanced Medical Sciences, Tokushima University, Tokushima, Japan.
6. Clinical Research Center for Diabetes, Tokushima University Hospital, Tokushima, Japan
7. Department of Diabetes and Molecular Genetics, Ehime University Graduate School of Medicine, Ehime, Japan.
8. **Reactivity of clone 38 to adiponectin multimers and monomer**

**Methods**

**Antibodies and antigens**

Two antibodies which detect the globular domain of human adiponectin, monoclonal mouse anti-human Adiponectin/Acrp30 antibody (Product code: MAB10651, Clone: 166126, Antibody Registry: AB_2221612) and monoclonal mouse anti-human Adiponectin/Acrp30 antibody (Product code: MAB1065, Clone: 166128, Antibody Registry: AB_2273512), and monoclonal mouse anti-human Adiponectin/Acrp30 antibody (Clone: 38, Sysmex, Hyogo, Japan) were employed to measure adiponectin in the antibody pairs shown in Table S1.

**Table S1.** Antibody clone names in three pairs employed to measure adiponectin.

|  | Capture Ab | Detection Ab |
| --- | --- | --- |
| Pair 1 | 166126 | 166128 |
| Pair 2 | 166128 | 38 |
| Pair 3 | 38 | 166128 |

**Preparation of capture antibody-coated magnetic particles**

The buffer was removed from 20 μL of streptavidin coated magnetic particles. 30 μL of capture antibody conjugate was added and incubated for 1 hour at 25 °C.

**Sandwich immunoassay for adiponectin**

The protocol of the sandwich immunoassay for adiponectin was as follows. 10 μL of sample was mixed with 100 μL of detection antibody conjugate and incubated for 1 min. After adding 30μL of capture antibody-coated magnetic particles, immune complexes were captured onto the surface of the beads during 13.5 min of incubation. All reactions up to this point were performed at 37°C. The bead was then washed, and the bound alkaline phosphatase activity was assayed by chemiluminescence with HISCL substrate reagent set (Sysmex, Hyogo, Japan) for 5.0 min at 42 °C. All reactions were performed automatically using a high-sensitivity immunoassay system, the HI-1000 (Sysmex, Hyogo, Japan).

**Preparation of HMW, MMW, LMW, and monomer adiponectin from urine**

One urine sample (1.0 mL) was selected from diabetic subjects and separated by SEC using an AKTA explorer 10S (GE Healthcare, Tokyo, Japan) on a column of HiLoad 16/60 Superdex 200 prep grade (1.6 x 60 cm) (GE Healthcare). An aliquot of each fraction was used for T-AN and H-AN assays. According to the results shown in Fig.1 and 4, fraction numbers 47-49, 52-55, 60-62 and 83-85 were used as samples of HMW, MMW, LMW, and monomer adiponectin, respectively.

**Results and discussions**

**Reactivity of clone 38 to adiponectin multimers and monomer**

Adiponectin concentrations measured by sandwich immunoassays using three different antibody pairs are shown in Fig.S1. Antibody pair 1 using clone 166126 and clone 166128 detected all types of adiponectin, which suggested both of these 2 clones react to all adiponectin multimers and monomer. When clone 166128 was used with clone 38 in antibody pairs 2 and 3, the same levels or relatively lower signal were detected from HMW and MMW adiponectin fractions. On the other hand, the signals from LMW and monomer adiponectin fractions were extremely decreased, which suggested that clone 38 reacts to HMW and MMW adiponectin and does not react to LMW and monomer adiponectin.

**Figure S1.** Adiponectin concentrations of adiponectin multimers and monomer fractions measured by sandwich immunoassays using three different antibody pairs.

1. **Additional data**

**Table S2.** Correlation between conventional biomarkers and T-AN or H-AN in the urine. (42-64 years old only)

|  |  |  | ND | DM | ND+DM |
| --- | --- | --- | --- | --- | --- |
| Albumin | T-AN | r | 0.21 | 0.61 | 0.47 |
|  |  | p | 0.15 | < 0.001 | < 0.001 |
|  | H-AN | r | 0.51 | 0.75 | 0.66 |
|  |  | p | 0.0002 | < 0.001 | < 0.001 |
| eGFR | Albumin | r | -0.032 | -0.32 | -0.16 |
|  |  | p | 0.83 | 0.044 | 0.14 |
|  | T-AN | r | 0.043 | -0.42 | -0.21 |
|  |  | p | 0.77 | 0.0062 | 0.046 |
|  | H-AN | r | -0.15 | -0.48 | -0.31 |
|  |  | p | 0.30 | 0.0016 | 0.0028 |
